# Testing for adaptive evolution of the female reproductive protein ZPC in mammals, birds and fishes reveals problems with the M7-M8 likelihood ratio test

**DOI:** 10.1186/1471-2148-5-65

**Published:** 2005-11-10

**Authors:** Sofia Berlin, Nick GC Smith

**Affiliations:** 1 Department of Evolutionary Biology, Evolutionary Biology Centre, Uppsala University, Norbyvagen 18 D, 752 36 Uppsala, Sweden; 2 Current address: Department of Genetics and Genomics, Roslin Institute (Edinburgh), Roslin, Midlothian EH25 9PS, UK; 3 Department of Mathematics and Statistics, Lancaster University, Lancaster LA1 4YF, UK

## Abstract

**Background:**

Adaptive evolution appears to be a common feature of reproductive proteins across a very wide range of organisms. A promising way of addressing the evolutionary forces responsible for this general phenomenon is to test for adaptive evolution in the same gene but among groups of species, which differ in their reproductive biology. One can then test evolutionary hypotheses by asking whether the variation in adaptive evolution is consistent with the variation in reproductive biology. We have attempted to apply this approach to the study of a female reproductive protein, zona pellucida C (ZPC), which has been previously shown by the use of likelihood ratio tests (LRTs) to be under positive selection in mammals.

**Results:**

We tested for evidence of adaptive evolution of ZPC in 15 mammalian species, in 11 avian species and in six fish species using three different LRTs (M1a-M2a, M7-M8, and M8a-M8). The only significant findings of adaptive evolution came from the M7-M8 test in mammals and fishes. Since LRTs of adaptive evolution may yield false positives in some situations, we examined the properties of the LRTs by several different simulation methods. When we simulated data to test the robustness of the LRTs, we found that the pattern of evolution in ZPC generates an excess of false positives for the M7-M8 LRT but not for the M1a-M2a or M8a-M8 LRTs. This bias is strong enough to have generated the significant M7-M8 results for mammals and fishes.

**Conclusion:**

We conclude that there is no strong evidence for adaptive evolution of ZPC in any of the vertebrate groups we studied, and that the M7-M8 LRT can be biased towards false inference of adaptive evolution by certain patterns of non-adaptive evolution.

## Background

Genes involved in reproduction and fertilization tend to evolve at faster rates than non-reproductive genes and it has been proposed that this rapid evolution is driven by positive Darwinian selection [1]. Mammalian sperm proteins have been extensively studied and they often show rapid divergence between closely related species [2-6]. The evolution of female reproductive proteins in vertebrate species has however received less attention, although evidence of positive selection has been provided for some female reproductive genes [7]. The precise nature of the selective pressures responsible for the accelerated evolution of reproductive genes are unknown, although plausible candidates include sexual selection, sexual conflict, and speciation reinforcement [1,8]. All three candidate evolutionary processes involve male-female interactions potentially manifested as sperm-egg interactions. So a way to test for these evolutionary processes is to compare patterns of evolution in genes involved in sperm-egg binding in (groups of) species, which differ with respect to sexual selection, sexual conflict or speciation reinforcement. For instance, in species with external fertilization, species recognition at the sperm-egg level should be more important than in species with internal fertilization, for which pre-fertilization mechanisms exist to avoid hybridisation. So if positive selection on reproductive genes is due to species recognition and hybridisation avoidance, then the signal of positive selection should be stronger in species with external fertilization than in species with internal fertilization. Furthermore, if species recognition at the sperm-egg level is driving the evolution of the genes involved in sperm-egg binding, then highly species-specific binding of sperm to the egg should yield a stronger signal of positive selection than less species-specific binding.

As an illustration of this general approach for determining the nature of positive selection in reproductive genes, we have investigated the molecular evolution of the gene encoding ZPC, the female reproductive glycoprotein zona pellucida C, in the three vertebrate groups of mammals, birds and fishes. The surface of the vertebrate oocyte is covered with an egg envelope, which is composed of several zona pellucida glycoproteins. ZPC is involved in the binding of sperm to the egg in mammals and birds, after which the acrosome reaction is triggered [9,10]. In general, when egg and sperm come from different mammalian species, binding of sperm to the egg envelope does not occur, thus the binding is species-specific [9]. From studies of sperm-egg binding in species from the avian order Galliformes (chicken and related species) it appears that the binding is not species-specific [11]. As a consequence, sperm-egg interactions in birds do not represent such a stringent species-specific barrier as they do in mammals. The fertilization mechanism in fish is rather different from that in mammals and birds. Most fish sperm lack an acrosome and penetrate the egg envelope via a discrete micropyle [12]. The function of the fish ZPC homologue is not entirely known, but studies in medaka (*Oryzias latipes*) suggest a function in guiding sperm to the micropyle, but it is unknown whether this process is species-specific.

In mammals, ZPC has previously been shown to be evolving by positive selection [7,13]. By considering the evolution of ZPC in birds and fishes as well as mammals we hoped to uncover variation in the levels of adaptive evolution among the three vertebrate groups. Since the three groups differ in their fundamental reproductive biology, any differences in adaptive evolution should be informative with regard to the underlying evolutionary mechanisms. By combining sequences from public databases and our own sequencing efforts, we constructed separate alignments of the ZPC coding sequence for 15 mammalian species, 11 avian species and six fish species. Our principle method for inferring adaptive evolution from ZPC sequence data was to apply LRTs of codon based models, which allow among-site variation in selection pressures. These models permit the inference of positive selection at a small proportion of sites, so that the signal of positive selection is not swamped by the (usually) much larger proportion of sites which are neutral or under negative selection. Such LRTs were used to demonstrate adaptive evolution of ZPC in mammals. However, there has been recent concern over high rates of false inference of positive selection with LRTs, particularly when applied to relatively small alignments with little sequence divergence [14-17]. Therefore we used three different LRTs and checked the evidence for positive selection by simulation studies.

## Results

### Overview of ZPC divergence

Using public available sequences and our own sequencing efforts, we produced three separate multiple species alignments of ZPC coding sequences for 15 mammals, 11 birds and six fishes. The total lengths of the alignments, which do not include start or stop codons but which do include gaps, are 1311 bp (437 codons) for mammals, 1335 bp (445 codons) for birds, and 1494 bp (498 codons) for fishes. Total tree length in substitutions per codon is 4.49 for mammals, 0.69 for birds, 4.49 for fishes. Thus there is relatively little sequence divergence in our sample of bird species compared to the other two groups of vertebrates.

We first analysed the ZPC interspecies alignments assuming the M0 model of no variation in ω among codons. ω is the nonsynonymous to synonymous rate ratio, also known as Ka/Ks or Dn/Ds, and is widely used as a measure of selective pressures on proteins assuming synonymous neutrality: ω<1 indicates negative selection, ω = 1 indicates neutrality, and ω>1 indicates positive selection. Average ω is 0.26 for mammals, 0.12 for birds and 0.32 for fishes. These low values indicate that the predominant mode of selection on the ZPC is purifying selection, and are consistent with the finding that domains within the ZPC gene are very well conserved across vertebrates [18]. Thus the M0 model of no variation in ω among codons gives no indication that ZPC is evolving unusually rapidly at the protein level.

### Likelihood ratio tests of positive selection

LRTs of positive selection compare the fit of two nested models to the sequence data; a null model without adaptive evolution and an alternative model with adaptive evolution. Both models may invoke variation in ω among codons, but the null model is restricted to ω less than or equal to one, whereas the alternative model allows adaptive evolution with ω>1. If the alternative model provides a significantly better fit to the data then adaptive evolution is inferred. We considered three different LRTs, three different pairs of null and alternative models: M1a-M2a, M7-M8, M8a-M8 (see Methods).

Table 1 provides a summary of all the LRTs of positive selection performed on the multiple species alignments of ZPC coding sequences of mammals, birds and fishes. Let us first consider the evolution of ZPC in mammals, since M7-M8 LRTs have previously been used to demonstrate positive selection across a wide range of mammals [7] and more specifically in the *Mus *genus [13]. There is no indication of positive selection using the M1a-M2a test, since no sites are inferred to be under positive selection in the M2a model (hence p = 1). In contrast, the M8 model indicates that 9.7% of codons are subject to weak positive selection (ω = 1.32), with the chi-square approximation to the LRT (see Methods) indicating a significant improvement in fit from M7 to M8 (p = 0.004) and a suggestive improvement in fit from M8a to M8 (p = 0.084).

**Table 1:** Summary of PAML analyses of ZPC multispecies alignments. Significant results are in bold.

	Mammals	Fishes	Birds
M0	ω = 0.26	ω = 0.32	ω = 0.12
M1a estimates	P1 = 0.71 ω1 = 0.096	P1 = 0.69 w1 = 0.16	P1 = 0.93 ω1 = 0.07
	P2 = 0.29 ω2 = 1.0	P2 = 0.31 w2 = 1.0	P2 = 0.07 ω2 = 1.0
M2a estimates	P1 = 0.71 ω1 = 0.096	P1 = 0.70 w1 = 0.16	P1 = 0.95 ω1 = 0.07
	P2+P3 = 0.29 ω2 = ω3 = 1.0	P2 = 0.0 ω2 = 1.0	P2 = 0.0 ω2 = 1.0
		P3 = 0.30 ω3 = 1.04	P3 = 0.053 ω3 = 1.27
2Δlogl(M1a-M2a)	0	0.07	0.28
M1a-M2a p(X^2^)	1	0.97	0.87
M8a estimates	P(beta) = 0.86	P(beta) = 0.72	P(beta) = 0.94
P1 = 0.16 ω1 = 1.0	P1 = 0.28 ω1 = 1.0	P1 = 0.065 ω1 = 1.0	
M8 positive selection	P = 0.097 ω = 1.32	P = 0.24 ω = 1.16	P = 0.049 ω = 1.29
2Δlogl(M7-M8)	10.9	14.9	3.67
M7-M8 p(X^2^)	**0.004**	**0.0006**	0.16
2Δlogl(M8a-M8)	3.00	0.75	0.32
M8a-M8 p(X^2^)	0.084	0.19	0.58

For birds, inferences of rare and weak positive selection are obtained for both the M2a (5.3% at ω = 1.27) and M8 (4.9% at ω = 1.29) models, although the chi-square approximations indicate no significant improvement in fits for alternative over null models (M1a-M2a p = 0.87, M7-M8 p = 0.16, M8a-M8 p = 0.58).

For fishes, the M2a model gives very little indication of positive selection (30% of codons with ω = 1.04), and the tiny increase in likelihood from M1a to M2a is not remotely significant (p = 0.97). The M8 model indicates that 24% of codons are subject to moderate positive selection (ω = 1.16). As with mammals the chi-square approximation indicates a significant improvement in fit for the M8 model over the M7 model (p = 0.0006). For the M8a-M8 comparison, the chi-square approximation indicates a non-significant improvement in fit (p = 0.19).

### Simulation studies

The LRT analyses show that the three different LRTs give rather different views of positive selection in ZPC. The M1a-M2a test gives no indication of adaptive evolution in any of the three vertebrate groups. The situation is similar for the M8a-M8 test, although the result in mammals is at least suggestive. In contrast, the M7-M8 test gives highly significant evidence of adaptive evolution in mammals and fishes, and even though the M7-M8 LRT is not significant in birds the M7-M8 test gives the lowest p value of the three tests.

How can we explain the differences among the LRTs? There are a number of possible explanations: (1) there is no adaptive evolution in ZPC in vertebrates, and the chi-square approximation is biased to give false positives for the M7-M8 test (but not the other two LRTs); (2) there is no adaptive evolution in ZPC in vertebrates, and the M7-M8 test is biased to give false positives because it is not robust to the complex patterns of non-adaptive evolution in ZPC (but not the other two LRTs); and (3) there is adaptive evolution, and the M7-M8 test has greater power to reveal adaptive evolution than the other two tests. We performed simulations to examine these alternative explanations.

To test explanation (1) we performed parametric bootstrapping to check the chi-square approximation to the likelihood ratio statistic (see Materials and Methods). We performed parametric bootstrapping with 100 simulated data sets for the three LRTs in both mammals and fishes. In each case we found a good correspondence between the p value for the null model obtained by parametric bootstrapping and the p value obtained by the chi-square approximation (see Table 2). There seems to be no reason to suggest that any biases in the chi-square approximation could have generated the M7-M8 evidence of adaptive evolution, i.e. the p values obtained by parametric bootstrapping for the M7-M8 tests remain significant.

**Table 2:** Summary of simulation studies of ZPC LRTs.

	p (X^2^)	mammals p (parametric bootstrap)	p (robustness)	p (X^2^)	fishes p (parametric bootstrap)	p (robustness)
M1a-M2a	1	100/100	100/100	0.97	57/100	32/100
M7-M8	0.004	<1/100	25/100	0.0006	<1/100	52/100
M8a-M8	0.084	7/100	7/100	0.19	28/100	28/100

To test explanation (2) we performed simulations to check the robustness of the three LRTs to complex patterns of non-adaptive evolution as captured by the M8a model (see Materials and Methods). The p values for the robustness simulations in Table 2 indicate the probability of obtaining the observed LRT results where the data generated under the M8a model. If the LRTs are unbiased then this p value should be similar to the chi-square p value, but if a LRT is biased to give false positives then we would expect an increase from the chi-square p value to the robustness p value. We find strong evidence of a bias affecting the M7-M8 LRT: for both mammals and fishes the observed findings of positive selection could easily have been generated by the non-adaptive M8a model of codon evolution. In contrast the M8a-M8 test seems unbiased while the M1a-M2a test appears a little conservative.

To test explanation (3) we looked at the relative powers of the LRTs using simulations with a weakly selected positive selection class (ω distribution of 40% ω = 0, 40% ω = 0.25, 10% ω = 1.0 and 10% ω = 1.5). Power was measured as the proportion of the 100 data sets for which the LRT, as assessed by the chi-square approximation, gave a significant result with p < 0.05. The power of the M1a-M2a test was only 12%, much lower than the 54% power achieved by the M8a-M8 test, while the M7-M8 test has the greatest power of 88%.

### Tests of positive selection in birds using polymorphism data

The fact that the total tree length of the avian multiple species alignment is relatively small means that methods using just inter-species divergence to infer positive selection are expected to lack power [14]. Therefore to study the evolution of avian ZPC in more detail we also performed various tests of positive selection making use of additional polymorphism data. The total number of segregating sites in the 46 chicken chromosomes was 56, of which 34 were intronic and 21 exonic, of which 3 were nonsynonymous and 18 synonymous. Tests based on allele frequency spectra failed to show significant deviations from neutrality or any evidence of recent selective sweeps. Tajima's *D *statistic was non-significantly positive (*D *= 0.074, p = 0.57), while the H test revealed a non-significant excess of high frequency alleles (*H *= -2.30, p = 0.13). The HKA test was performed to test for heterogeneity between the ratio intraspecific variation in chicken and interspecific divergence with the turkey outgroup in ZPC against a reference set of autosomal data from Sundstrom et al. [19].

The reference set contained only intronic sites, so only intronic sites in ZPC were considered. The HKA test did not show significant deviation from neutrality (p = 0.60). The nucleotide diversity in ZPC introns, θ_π_, is 8.9 × 10^-3^, higher by a factor of 1.37 than the average for several other intronic loci sequenced in the same individuals (θ_π _= 6.5 × 10^-3^) [19]. The relative increase in polymorphism is very nearly matched by the relative increase in divergence: the ZPC chicken-turkey intronic divergence of 0.129 is higher by a factor of 1.40 than the average of 0.092 from the study of Sundstrom et al. [19]. The McDonald-Kreitman test was performed to compare the nonsynonymous over synonymous ratio between divergence and polymorphism. For chicken polymorphism there were 3 nonsynonymous and 18 synonymous changes, while for divergence the numbers of nonsynonymous and synonymous changes down the chicken lineage were estimated by PAML to be 12 and 28. Thus there is a signal of positive selection, i.e. a relative excess of nonsynonymous divergence, but it is not significant (Fisher's exact test 1-T, p = 0.15).

## Discussion

We have tested for signals of positive selection in the evolution of the ZPC gene in mammals, birds and fishes. The three different LRTs yielded some conflicting results (see Table 1), with the only two significant findings of positive selection both obtained using the M7-M8 test. The parametric bootstrapping simulations showed that this discrepancy between the LRTs was not due to biases in the chi-square approximation to the likelihood ratio statistic. The power simulations showed that the M7-M8 test is more powerful at detecting low levels of weak positive selection than the two other LRTs (as shown in [17]), but the robustness simulations showed that the M7-M8 test is biased towards false positives under patterns of non-adaptive evolution like those fitted by the M8a model to the ZPC gene. The bias in the M7-M8 test revealed by the robustness simulations was not apparent for the M1a-M2a and M8a-M8 tests, and this difference seems to provide the most likely explanation for our results: the highly significant M7-M8 results could have been generated by the bias in the test in the absence of adaptive evolution.

Thus we have no strong evidence of adaptive evolution in ZPC in vertebrates, with the best evidence being the suggestive p value of 0.08 for the M8a-M8 LRT on the mammalian sequence data. The use of avian polymorphism data in addition to interspecies data also failed to reveal significant evidence of positive selection.

Our results have some important methodological implications. The potential bias of the M7-M8 test has been suggested as a theoretical possibility by Swanson et al. [4], who pointed out that if the beta distribution on its own (the M7 model) fits the data poorly, then the M7-M8 test may generate a high proportion of significant tests even in the absence of positive selection. As far as we know, our robustness simulations provide the first demonstration of the M7-M8 bias for real data. The beta distribution is a natural distribution for modelling the variation in ω between 0 and 1 (e.g. see figures in the PAML manual), but a single beta distribution cannot provide a good fit when the real data have a bimodal distribution with two peaks, one located at ω = 1 and the other at an intermediate ω above 0 but less than 0.5. Thus the addition of an extra ω class in the M8 model is likely to give a large increase in likelihood, and if the position of the ω = 1 peak is overestimated at all then positive selection will be inferred.

We tested these ideas of why the M7-M8 LRT can lead to an excess of false positives by performing some additional robustness simulations under simple codon models. In all other respects except the ω distribution our set of 100 replicates was generated with the same parameters as the 5-taxon tree datasets detailed in Wong et al. [17]. The simulated ω distribution was 40% ω = 0, 40% ω = 0.25 and 20% ω = 1.0. In 18 out of 100 replicates the M7-M8 test gave a false positive result at the 5% level using the chi-square method, and six replicates gave a false positive result at the 1% level. So these robustness simulations confirm that the M7-M8 test can be biased under fairly simple scenarios. Since the scenario we have simulated seems biologically reasonable, i.e. a combination of some sites under very strong negative selection, some sites under moderate negative selection, and a small proportion of sites evolving neutrally, we believe that evidence of adaptive evolution obtained with the M7-M8 LRT alone should be treated with caution.

We also considered the performance of the M1a-M2a and M8a-M8 LRTs for the same simulated data, which led to an excess of false positives with the M7-M8 test. In none of the 100 replicates did the M1a-M2a test give a false positive result at the 5% level, indicating a conservative test. For the M8a-M8 test the levels of false positives were slightly elevated above expectations with nine false positives at the 5% level and two false positives at the 1% level, but these levels are reasonably close to null expectations. Thus the choice between the M1a-M2a and M8a-M8 LRTs can be seen as a tradeoff: the M8a-M8 test has greater power but also a greater risk of false positives.

We should emphasize the importance of distinguishing between problems with specific LRTs and the general approach using LRTs to test for adaptive evolution by comparing the likelihoods of different models of ω variation among codons. Our robustness simulations indicate that the M7-M8 LRT tends to produce false positives in the absence of adaptive evolution. Such a failure of a specific LRT does not mean that the general approach of LRTs is wrong, just that certain LRTs may fail under certain conditions. The more we know about how genes evolve the better we will be able to design models of among site variation in ω.

One final methodological point concerns the effects of changes in the size of the dataset. In a preliminary version of this study we considered a more limited dataset of just four fish species. For this dataset we obtained significant results of p = 0.044 for the M1a-M2a LRT and p = 0.032 for the M8a-M8 LRT. The addition of two more species (*Carassius auratus *and *Pimephales promelas*) increased the M1a-M2a p value to 0.97 and the M8a-M8 p value to 0.19. These two species only increased total length slightly from 4.14 to 4.49, so it is surprising that they had such a large effect on the M8a-M8 test. It seems likely that small datasets are likely to lead imprecise maximum likelihood estimates of parameters, particularly when fitting parameter rich codon models. Such concerns may be addressed by using Bayesian approaches to the inference of adaptive evolution, [20,21] which should account for parameter uncertainty.

## Conclusion

So as far as biological conclusions go, we have no strong evidence of adaptive evolution in ZPC in vertebrates. This result obviously means that we cannot use comparative methods to analyse the selective causes of adaptive evolution in ZPC. Our study does however raise some interesting methodological issues, in particular we show that the patterns of evolution in the ZPC gene cause the M7-M8 LRT to be heavily biased towards false inference of adaptive evolution. Thus we urge caution in the use of the M7-M8 LRT, and would suggest the M1a-M2a and M8a-M8 LRTs as more reliable tests of adaptive evolution. Much of the evidence for pervasive adaptive evolution in reproductive proteins has been obtained using the M7-M8 LRT and our study raises the issue of whether these findings are genuine.

## Methods

### Sequencing of avian ZPC sequences

We have included eleven bird species from the order Galliformes in this study: black grouse (*Tetrao tetrix*), chicken (*Gallus gallus*), grey partridge (*Perdix perdix*), hazel grouse (*Bonasa bonasia*), pheasant (*Phasianus colchicus*), ptarmigan (*Lagopus mutus*), quail (*Coturnix coturnix*), red grouse (*Lagopus lagopus*), red-legged partridge (*Alectoris rufa*), sage grouse (*Centrocercus urophasianus*) and turkey (*Meleagris gallopavo*). In addition, we sequenced 23 male chickens. For information about these individuals see Sundstrom et al. [19]. The *ZPC *gene is located on chromosome 10 in chicken, according to the UCSC chicken genome browser. The gene has been fully sequenced in chicken [Genbank:AB031033], hence the exon-intron boundaries are known. We intended to sequence the entire gene, including exons and introns for all eleven species and the 23 male chickens. Despite many efforts, certain regions (nucleotides 1–373 and 715–773 in grey partridge, 315–372 and 411–445 in hazel grouse, 915–1007 in red-legged partridge and 414–442 and 790–815 in ptarmigan) could not be PCR amplified and were therefore not analyzed in these species.

We PCR amplified the gene from genomic DNA in five overlapping fragments using the following primer combinations: ZPC1051F/ZPCe2R, ZPCe2F/ZPCe5R, ZPCe4F/ZPCe6R, ZPC5F/ZPC7R and ZPC7F/ZPC9R (Primer sequences, primer positions and PCR conditions available on request). The same primers were used for all species except for ZPCe1Fmel, which was used together with ZPCe2R to amplify fragment 1 in turkey. The PCR reactions were performed in 20 μl volumes on Perkin Elmer 9600 Thermal Cyclers using 0,5 U AmpliTaq Gold (Applied Biosystems), 1.9–3 mM MgCl_2 _(Applied Biosystems) (exact concentrations avalailable on request), 0.08 mM dNTPs, 1x PCR Gold Buffer (Applied Biosystems), 5 pmol of each primer and 50 ng of template DNA. Turkey fragment 1 was amplified using Hotstar polymerase (Qiagen). PCR products were separated on 2% agarose gels, run in 0.5% TAE buffer, and visualized by ethidium bromide staining. PCR products were, prior to sequencing, purified with ExoSAP-IT reagent (Amersham Biosciences) followed by direct sequencing in forward and reverse directions using the DYEnamic™ ET DyeTerminator Kit (Amersham Biosciences). Sequencing primer sequences are available on request. Reactions were electrophoresed on a MegaBACE 1000 sequencing instrument (Amersham Bioscences). The sequences were edited in Autoassembler (Applied Biosystems) and overlapping forward and reverse sequences were compared to make consensus sequences. Complete diploid sequences from the 23 chicken individuals were assembled using ambiguity codes at heterozygote positions. Sequences have been deposited in public sequence databases [Genbank:AY628608-AY628630, Genbank:AY630568-AY630572, GenBank:DQ004565-DQ004569].

### ZPC sequences of mammals and fishes

We collected a total of 15 mammalian ZPC sequences with accessions as follows: [Genbank:M20026] (mouse, *Mus musculus*), [Genbank:Y10823] (rat, *Rattus rattus*), [Genbank:X56777] (human, *Homo sapiens*), [Genbank:S71825] (marmoset), [Genbank:X82639] (macaque, *Macaca radiata*), [Genbank:D45070] (dog, *Canis familiaris*), [Genbank:D45068] (cat, *Felis catus*), [Genbank:D45065] (pig, *Sus scrofa*), [Genbank:NM_173974] (cow, *Bos taurus*), [Genbank:U05782] (rabbit, *Oryctolagus cuniculus*), [Genbank:AY598032] (fox, *Vulpes vulpes*), [Genbank:AY702973] (ferret, *Mustela putorius*), [Genbank:AF515621] (steppe lemming, *Lagarus lagurus*), [Genbank:AF304487] (Brandt's vole, *Microtus brandti*), [Genbank:M63629] (golden hamster, *Mesocricetus auratus*). The first eight sequences were taken from the analysis by Swanson et al. [7], while the remaining seven sequences were identified as homologs by BLAST searches [22], confirmed by annotation, and chosen for their effects on total branch length.

We collected six fish ZPC sequences with accessions as follows: [Genbank:NM_131331] (zebrafish, *Danio rerio*), [Genbank:L41638] (common carp, *Cyprinus carpio*), [Genbank:AF231708] (rainbow trout, *Oncorhynchus mykiss*), [Genbank:D38630] (Japanese medaka, *Oryzias latipes*), [Genbank:Z48974] (goldfish, *Carassius auratus*), and [Genbank:AF192407] (fathead minnow, *Pimephales promelas*). The first five sequences were confirmed as ZPC orthologs by Spargo and Hope [23], and the final sequence was identified as a homolog by a BLAST search and confirmed by annotation.

### Construction of multiple species alignments

All alignments were generated using CLUSTALW [24]. The coding sequence alignments of DNA sequences were based on the protein alignments. Before we aligned the avian ZPC introns we removed regions of simple repeats, identified by the program Sputnik [25] since they make alignments unreliable and are a potential cause of bias due to elevated substitution and indel rates.

### Likelihood ratio tests of positive selection

We used the codeml program in the PAML package [26] version 3.14 to perform likelihood ratio tests of positive selection. We considered models of codon evolution which allow for variation in ω among codons but assume the same distribution in all lineages. Likelihood ratio tests (LRTs) compare the maximum likelihoods of pairs of nested models, and when the models differ in whether they include codons under positive selection with ω>1, then the LRT is a test for positive selection [27,28]. There are many possible models of ω variation among codons, and hence many possible LRTs of positive selection. We performed three LRTs, which are thought to provide reliable tests of positive selection [4,17]. M1a-M2a LRT: The M1a model (one ω class between 0 and 1, and one class of ω = 1) is compared to the M2a model (same as M1a model plus an extra class of ω>1). M7-M8 LRT: The M7 model (a discretised beta distribution for ω between 0 and 1 with 10 equal class proportions) is compared to the M8 model (same as the M7 model plus an extra class of ω≥1). M8a-M8 LRT: The M8a model (same as M7 plus an extra class of ω = 1) is compared to the M8 model. For all LRTs, the first model is a simplified version of the second, with fewer parameters, and is thus expected to provide a poorer fit to the data (lower maximum likelihood). The first model is the null model without adaptive evolution and the second model is the alternative model with adaptive evolution, so a significant improvement in maximum likelihood supports positive selection.

The significance of likelihood ratio tests is usually calculated using the chi-square approximation, which states that at the asymptote when there is a large amount of data, then twice the difference in the log of maximum likelihood between the two models (the likelihood ratio statistic 2Δlogl) is distributed as a chi-square distribution with the degrees of freedom (df) given by the difference in the numbers of parameters in the two nested models. For the M1a-M2a comparison df = 2. For the M7-M8 and M8a-M8 comparisons the situation is more complicated due to problems with non-estimable parameters and parameter values being bounded. For the M7-M8 comparison the use of df = 2 is expected to be conservative, and for the M8a-M8 comparison the use of df = 1 is expected to be conservative.

For all LRTs, equilibrium codon frequencies were obtained using the average base composition at the three codon positions (CodonFreq = 2) and the transition-transversion rate ratio was estimated from the data. For the analysis of mammal and fish alignments of complete sequences, sites with ambiguity data were removed (cleandata = 1) since they correspond to indels. But for the analysis of the bird alignments, for which the sequences of some species were incompletely sequenced (see above), we did not remove sites with ambiguity data (cleandata = 0). One problem with the implementation of LRTs is the existence of suboptimal local maxima, which is overcome by use of several different starting points for the likelihood maximization. We checked carefully for local maxima in our analyses of real data, but not in our analyses of simulated data. We know that the PAML search algorithm did occasionally stall at suboptimal local optima in the analyses of our simulated data, since in around 1–2% of cases we obtained a higher likelihood with the simpler model, but such a low proportion is only expected to cause a slight downward bias to our simulation p values.

### Simulation studies of LRTs

There are two potential problems with LRTs, which may generate false positives, i.e. the inference of adaptive evolution when there is none. (1) The first potential problem is that the chi-square approximation to the likelihood ratio statistic may be poor when data are limited. The problem in this case is not that the test itself is biased, but that the standard method for assessing significance of the likelihood ratio statistic does not hold. (2) The second potential problem is that certain LRTs may not be robust to certain patterns of non-adaptive evolution, i.e. they may have high rates of false positives. This case represents the more serious problem, that the LRT is inherently flawed in that it indicates positive selection even when there is non present. Both problems (1) and (2) were addressed by simulation studies performed using the evolver program in the PAML package.

We investigated the problem (1) by empirical parametric bootstrapping. For each LRT data was simulated using the maximum likelihood estimates of the parameters of the null model (M1a for M1a-M2a, M7 for M7-M8 and M8a for M8a-M8). The simulated data were then analysed using both the null and alternative models, and these results were used to generate the null distribution of the likelihood ratio statistic. Comparison of the likelihood ratio obtained from the real sequences with the null distribution of the likelihood ratios obtained from the simulated sequences indicates whether the null model of non-adaptive evolution is rejected in favour of the alternative model.

We addressed problem (2) by robustness simulations. We simulated data sets using the maximum likelihood parameter estimates obtained for the M8a model. Thus we simulated sequence data according to the richest model without positive selection, irrespective of which LRT was to be used to analyse the data. The simulated data were analysed using both the null and alternative models specific to each LRT, and these results were used to generate the null distribution of the likelihood ratio statistic. Comparison of the likelihood ratio obtained from the real sequences with the null distribution of the likelihood ratios obtained from the simulated sequences indicates whether the observed results could have been generated by non-adaptive evolution.

### Phylogenetic trees

**Figure 1:**
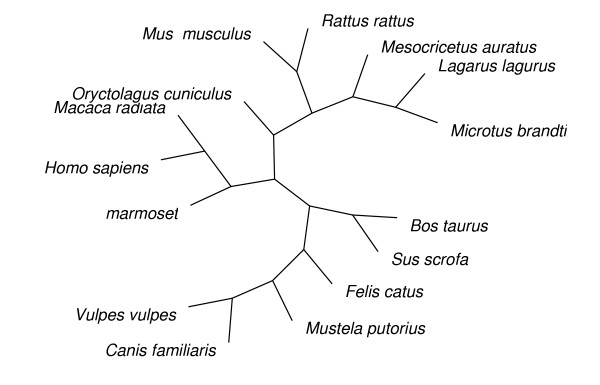
The unrooted mammal tree used for the PAML analyses.

**Figure 2:**
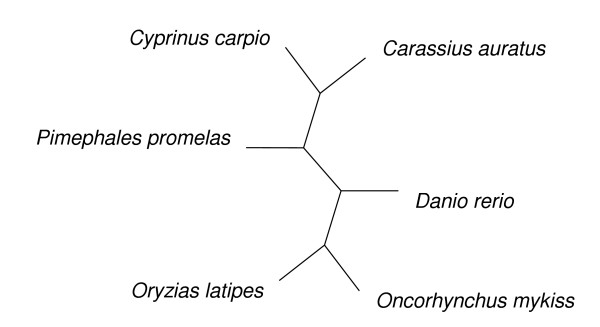
The unrooted fish tree used for the PAML analyses.

**Figure 3:**
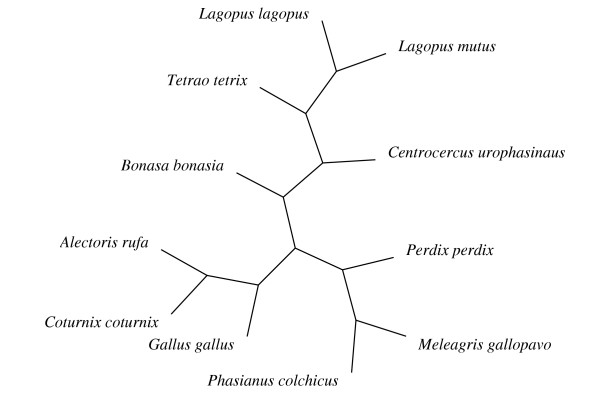
The unrooted bird tree used for the PAML analyses.

The PAML analyses require a single unrooted phylogenetic tree. The trees used in our analyses are shown in Figures 1 to 3. The mammalian tree is consistent with the emerging molecular view of mammalian phylogeny [29]. The fish tree is consistent with the tree in Spargo and Hope [23] for all species except *Pimephales promelas*, which was inferred to group with the other species in the Cyprinidae family using the stepwise addition method in PAML under the M0 model of no variation in ω among codons. Since Galliformes phylogeny is relatively uncertain we used MrBayes version 3.0b4 [30] to analyse the bird intron alignments. We specified a general time reversible model of nucleotide evolution, with a proportion of sites invariable and a gamma rate distribution for the remaining sites (using the MrBayes command "lset nst = 6 rates = invgamma"). The consensus bird tree we obtained differs slightly from the inferred phylogenies of Dimcheff et al. [31], but the differences are at nodes with relatively low bootstrap support in Dimcheff et al.'s maximum likelihood analysis of their mitochondrial sequences, and when we repeated our PAML analyses with a tree corresponding to the results of Dimcheff et al. we found no qualitative change to our results (data not shown).

### Tests of positive selection using polymorphism data

We used DnaSP 4.0 [32] to analyse patterns of polymorphism in our chicken ZPC sequences. McDonald-Kreitman tests [33] were performed with polymorphism in exons inferred using DnaSP and divergence estimated using PAML. The HKA test [34] was performed using the HKA computer program written by Jody Hey [35]. Intronic divergence was estimated using the baseml program in the PAML package under the Tamura-Nei model of nucleotide substitution [36]. We performed tests of selective sweeps using Tajima's D statistic [37] and the *H *statistic [38] using a computer program written by Justin Fay [39]. In order to infer the frequency of polymorphisms in chicken (required for the H test) we used parsimony with the turkey sequence as outgroup. Polymorphisms potentially due to CpG hypermutability were removed since parsimony inference may be poor in such cases. The probability of misinference was calculated as suggested by Fay and Wu [38] using the average intronic divergence between chicken and turkey (maximum likelihood distance of 0.129 given by PAML). The population scaled measure of recombination was estimated to be 65 across the whole gene using PHASE version 2.1 [40,41] to estimate a constant recombination rate with the unphased chicken data.

## Authors' contributions

SB conceived the study and performed DNA sequencing of bird samples. NGCS performed the likelihood ratio test studies. Both authors designed the study, participated in sequence analyses of bird sequences, drafted the manuscript, and read and approved the final edition. Just after this manuscript was submitted Nick Smith tragically died in an accident. Nick was a splendid scientist and a great source of inspiration to me. He will be greatly missed.
